# Supplementary material for: Comprehensive multi-omics analysis reveals prognostic, immune, and therapeutic signatures of TNFAIP family genes in breast cancer
Source: PLoS One. 2026 May 29;21(5):e0349012. doi: 10.1371/journal.pone.0349012 (PMC13221070; doi:10.1371/journal.pone.0349012)
Supplement: S1 Table — (DOCX) [file pone.0349012.s001.docx]

**S1 Table** | Key regulated TFs of TNFAIP family in BC

| Key TFF | Description | Regulated gene | P.value | Adj P-value |
| --- | --- | --- | --- | --- |
| NR3C1 | Nuclear Receptor Subfamily 3 Group C Member 1 | EFNA1; TNFAIP8; STEAP4; TNFAIP2; TNFAIP3; PTX3 | 0.0002826 | 0.06306 |
| RELA | RELA proto-oncogene, NF-kB subunit | TNFAIP2; TNFAIP3; PTX3; TNFAIP1 | 0.0005403 | 0.06306 |
| BACH1 | BTB Domain And CNC Homolog 1 | EFNA1; STEAP4; TNFAIP6; TNFAIP3 | 0.0005846 | 0.06306 |
| CEBPA | CCAAT Enhancer Binding Protein Alpha | STEAP4; TNFAIP6; TNFAIP3 | 0.0005977 | 0.06306 |
| NFKB1 | Nuclear Factor Kappa B Subunit 1 | EFNA1; TNFAIP8; STEAP4; TNFAIP6; TNFAIP2; TNFAIP3 | 0.0009531 | 0.08044 |
| CEBPD | CCAAT Enhancer Binding Protein Delta | TNFAIP8; TNFAIP6; TNFAIP3; PTX3 | 0.001157 | 0.08140 |
| CEBPB | CCAAT Enhancer Binding Protein Beta | STEAP4; TNFAIP6; TNFAIP3; PTX3 | 0.001863 | 0.08224 |
| AR | Androgen Receptor | EFNA1; STEAP4; TNFAIP2; TNFAIP3 | 0.001904 | 0.08224 |
| TAL1 | TAL BHLH Transcription Factor 1, Erythroid Differentiation | TNFAIP8; STEAP4; TNFAIP2; TNFAIP3 | 0.002042 | 0.08224 |
| TCF12 | Transcription Factor 12 | TNFAIP8; STEAP4; TNFAIP2; TNFAIP3 | 0.002161 | 0.08224 |
