# Supplementary material for: Comprehensive multi-omics analysis reveals prognostic, immune, and therapeutic signatures of TNFAIP family genes in breast cancer
Source: PLoS One. 2026 May 29;21(5):e0349012. doi: 10.1371/journal.pone.0349012 (PMC13221070; doi:10.1371/journal.pone.0349012)
Supplement: S2 Table — (DOCX) [file pone.0349012.s002.docx]

**S2 Table** | Key miRNAs regulating TNFAIPs in BC

| Key miRNA | Regulated gene | P.value | Adj P-value |
| --- | --- | --- | --- |
| hsa-miR-9-5p | EFNA1; TNFAIP8; PTX3 | 0.0002811 | 0.06317 |
| hsa-miR-19a-5p | TNFAIP6; TNFAIP3 | 0.0008066 | 0.06317 |
| hsa-miR-130a-5p | TNFAIP8; TNFAIP3 | 0.001162 | 0.06317 |
| hsa-miR-23c | TNFAIP8; TNFAIP3 | 0.001179 | 0.06317 |
| hsa-miR-654-5p | EFNA1; TNFAIP8 | 0.001704 | 0.06317 |
| hsa-miR-6769a-5p | EFNA1; TNFAIP8 | 0.001768 | 0.06317 |
| hsa-miR-541-3p | EFNA1; TNFAIP8 | 0.001855 | 0.06317 |
| hsa-miR-6769b-5p | EFNA1; TNFAIP8 | 0.001900 | 0.06317 |
| mmu-miR-665-3p | STEAP4; TNFAIP6 | 0.003229 | 0.09542 |
| hsa-miR-92a-2-5p | EFNA1; TNFAIP8 | 0.004050 | 0.09949 |
