# Supplementary material for: Comprehensive multi-omics analysis reveals prognostic, immune, and therapeutic signatures of TNFAIP family genes in breast cancer
Source: PLoS One. 2026 May 29;21(5):e0349012. doi: 10.1371/journal.pone.0349012 (PMC13221070; doi:10.1371/journal.pone.0349012)
Supplement: S3 Table — (DOCX) [file pone.0349012.s003.docx]

**S3 Table** | The prognostic significance of TNFAIP family single CpG methylation in patients with BC (MethSurv).

| Gene-CPG | HR | LR test p-value | FDR |
| --- | --- | --- | --- |
| TNFAIP1 - Body-Open_Sea-cg0926757 | 0.439 | 0.00014 | 0.00157 |
| TNFAIP1 - Body-Open_Sea-cg13290523 | 0.468 | 0.0017 | 0.0151 |
| TNFAIP1 - Body-Open_Sea-cg23245800 | 0.655 | 0.077 | 0.0953 |
| TNFAIP1 - 3’UTR-Open_Sea-cg15159588 | 2.582 | 0.012 | 0.074 |
| TNFAIP1 - Body-S_Shelf-cg15159588 | 0.686 | 0.075 | 0.0953 |
| TNFAIP2 - Body-Island-cg00731608 | 0.539 | 0.0019 | 0.0160 |
| TNFAIP2 - Body-Island-cg09814127 | 1.402 | 0.1 | 0.114 |
| TNFAIP2 - Body-Island-cg10501093 | 1.451 | 0.062 | 0.086 |
| TNFAIP2 - Body-Island-cg18587137 | 0.807 | 0.035 | 0.059 |
| TNFAIP2 - Body-Island-cg18620571 | 0.75 | 0.021 | 0.046 |
| TNFAIP2 - Body-N_Shelf-cg03572388 | 0.795 | 0.24 | 0.267 |
| TNFAIP2 - 5`UTR;1stExon-N_Shore-cg04264002 | 0.73 | 0.17 | 0.197 |
| TNFAIP2 – TSS200-N_Shore-cg05288750 | 1.444 | 0.11 | 0.124 |
| TNFAIP2 – TSS1500-N_Shore-cg05373539 | 0.632 | 0.031 | 0.054 |
| TNFAIP2 – TSS1500-N_Shore-cg16577823 | 0.477 | 0.0018 | 0.0160 |
| TNFAIP2 – Body-N_Shore-cg20368904 | 0.505 | 0.00094 | 0.0095 |
| TNFAIP2 – Body-Open_Sea-cg08301307 | 0.884 | 0.059 | 0.084 |
| TNFAIP2 – Body-Open_Sea-cg09274347 | 0.659 | 0.039 | 0.059 |
| TNFAIP2 - Body-S_Shelf-cg13144594 | 0.542 | 0.0037 | 0.035 |
| TNFAIP2 - Body-S_Shore-cg16127617 | 0.621 | 0.028 | 0.049 |
| TNFAIP3 - TSS200-Island-cg00847892 | 0.669 | 0.12 | 0.128 |
| TNFAIP3 - TSS200-Island-cg08667148 | 1.291 | 0.28 | 0.296 |
| TNFAIP3 - TSS200-Island-cg09763362 | 2.16 | 0.00014 | 0.00112 |
| TNFAIP3 - TSS200-Island-cg12214665 | 0.846 | 0.46 | 0.467 |
| TNFAIP3 - TSS200-Island-cg15380607 | 0.64 | 0.041 | 0.059 |
| TNFAIP3 – TSS1500-Island-cg05131254 | 1.394 | 0.16 | 0.178 |
| TNFAIP3 – TSS1500-Island-cg06779945 | 1.32 | 0.19 | 0.207 |
| TNFAIP3 – TSS1500-Island-cg22466620 | 1.857 | 0.45 | 0.467 |
| TNFAIP3 – TSS1500-Island-cg23696891 | 1.257 | 0.32 | 0.338 |
| TNFAIP3 - 5`UTR-Island-cg14527802 | 0.689 | 0.062 | 0.084 |
| TNFAIP3 - 5`UTR-Island-cg26960647 | 1.241 | 0.28 | 0.296 |
| TNFAIP3 - 5`UTR;1stExon-Island -cg18264753 | 1.307 | 0.21 | 0.228 |
| TNFAIP3 – Body-Open_Sea-cg01981433 | 0.653 | 0.038 | 0.057 |
| TNFAIP3 – Body-Open_Sea-cg11812071 | 0.664 | 0.038 | 0.057 |
| TNFAIP3 – Body-Open_Sea-cg12200164 | 0.701 | 0.16 | 0.178 |
| TNFAIP3 – Body-Open_Sea-cg18287768 | 1.638 | 0.025 | 0.038 |
| TNFAIP3 – Body-Open_Sea-cg19862242 | 1.461 | 0.0016 | 0.0179 |
| TNFAIP3 – Body-Open_Sea-cg25934495 | 0.782 | 0.26 | 0.276 |
| TNFAIP3 – 3`UTR-Open_Sea-cg05987705 | 0.675 | 0.057 | 0.079 |
| TNFAIP3 – 3`UTR-Open_Sea-cg12050508 | 0.795 | 0.25 | 0.267 |
| TNFAIP3 - 5`UTR-S_Shore-cg25971086 | 1.209 | 0.41 | 0.426 |
| TNFAIP3 - 5`UTR-S_Shore-cg27031112 | 0.729 | 0.11 | 0.124 |
| EFNA1 - 1stExon-S_Shore-cg03231024 | 0.684 | 0.0057 | 0.049 |
| EFNA1 - 1stExon-S_Shore-cg26809127 | 1.289 | 0.28 | 0.296 |
| EFNA1 - Body-S_Shore-cg07207669 | 1.569 | 0.032 | 0.049 |
| EFNA1 - 5`UTR;1stExon-Island -cg04296885 | 0.408 | 0.15 | 0.178 |
| EFNA1 - 5`UTR;1stExon-Island -cg24526499 | 1.598 | 0.058 | 0.084 |
| EFNA1 – TSS1500-Island-cg05082095 | 0.806 | 0.33 | 0.352 |
| EFNA1 – TSS1500-Island-cg05641351 | 1.336 | 0.19 | 0.207 |
| EFNA1 – TSS1500-Island-cg08250444 | 0.483 | 0.00035 | 0.0062 |
| EFNA1 – TSS1500-Island-cg10861146 | 1.13 | 0.59 | 0.615 |
| EFNA1 – TSS1500-Island-cg21609826 | 0.737 | 0.16 | 0.178 |
| EFNA1 – TSS1500-Island-cg25723933 | 0.742 | 0.15 | 0.178 |
| EFNA1 – TSS1500-Island-cg26667821 | 0.457 | 0.00034 | 0.0062 |
| EFNA1 - TSS200-Island-cg13298932 | 0.814 | 0.31 | 0.332 |
| EFNA1 - TSS200-Island-cg16273215 | 1.235 | 0.32 | 0.338 |
| EFNA1 - TSS200-Island-cg26335527 | 1.91 | 0.0037 | 0.037 |
| EFNA1 - Body-S_Shelf-cg06639488 | 0.89 | 0.56 | 0.615 |
| EFNA1 - Body-S_Shelf-cg14612733 | 0.777 | 0.28 | 0.296 |
| EFNA1 - Body-S_Shelf-cg16677112 | 0.656 | 0.035 | 0.051 |
| EFNA1 – Body-N_Shelf-cg12052789 | 0.595 | 0.0099 | 0.061 |
| EFNA1 – Body-N_Shelf-cg19628619 | 0.761 | 0.18 | 0.207 |
| TNFAIP6 - TSS1500-Open_Sea-cg01035238 | 0.817 | 0.37 | 0.392 |
| TNFAIP6 - TSS1500-Open_Sea-cg01189638 | 0.795 | 0.33 | 0.352 |
| TNFAIP6 - TSS1500-Open_Sea-cg01974138 | 1.403 | 0.096 | 0.108 |
| TNFAIP6 – TSS200-Open_Sea-cg01942558 | 0.444 | 0.00012 | 0.00112 |
| TNFAIP6 - Body-Open_Sea-cg03406844 | 0.467 | 0.00014 | 0.00124 |
| TNFAIP6 - 5`UTR;1stExon-Open_Sea-cg09727050 | 0.582 | 0.0076 | 0.054 |
| TNFAIP8 – Body-N_Shelf-cg00421693 | 1.296 | 0.19 | 0.207 |
| TNFAIP8 - Body-Open_Sea-cg00524900 | 0.638 | 0.026 | 0.046 |
| TNFAIP8 - Body-Open_Sea-cg01057573 | 0.495 | 0.0012 | 0.014 |
| TNFAIP8 - Body-Open_Sea-cg01915433 | 0.835 | 0.42 | 0.436 |
| TNFAIP8 - Body-Open_Sea-cg04195774 | 1.741 | 0.031 | 0.049 |
| TNFAIP8 - Body-Open_Sea-cg07398791 | 0.856 | 0.43 | 0.436 |
| TNFAIP8 - Body-Open_Sea-cg15408889 | 1.806 | 0.003 | 0.034 |
| TNFAIP8 - Body-Open_Sea-cg15621656 | 0.492 | 0.00065 | 0.0065 |
| TNFAIP8 - Body-Open_Sea-cg21239001 | 0.771 | 0.26 | 0.276 |
| TNFAIP8 – 3`UTR-Open_Sea-cg24957532 | 0.929 | 0.74 | 0.752 |
| TNFAIP8 - Body-S_Shore-cg00629563 | 0.6 | 0.011 | 0.064 |
| TNFAIP8 - Body-S_Shore-cg14692284 | 1.227 | 0.33 | 0.352 |
| TNFAIP8 - Body-1stExon-S_Shore-cg10911160 | 0.822 | 0.32 | 0.352 |
| TNFAIP8 – TSS1500-N_Shore-cg01810267 | 0.835 | 0.43 | 0.436 |
| TNFAIP8 – TSS1500-N_Shore-cg03723497 | 0.518 | 0.0035 | 0.038 |
| TNFAIP8 – TSS1500-N_Shore-cg07376834 | 0.909 | 0.71 | 0.752 |
| TNFAIP8 – TSS1500-N_Shore-cg18689486 | 1.099 | 0.68 | 0.752 |
| TNFAIP8 – TSS1500-N_Shore-cg21130861 | 0.811 | 0.37 | 0.392 |
| TNFAIP8 – Body-TSS1500-N_Shore-cg02283238 | 0.689 | 0.06 | 0.084 |
| TNFAIP8 – Body-TSS1500-N_Shore-cg07086380 | 0.65 | 0.031 | 0.051 |
| TNFAIP8 – Body-TSS1500-N_Shore-cg17471836 | 0.664 | 0.044 | 0.060 |
| TNFAIP8 – Body-TSS1500-N_Shore-cg23985447 | 1.072 | 0.72 | 0.752 |
| TNFAIP8 – Body-N_Shore-cg03665078 | 0.823 | 0.33 | 0.352 |
| TNFAIP8 – TSS200-N_Shore-cg04280246 | 0.592 | 0.01 | 0.057 |
| TNFAIP8 - 5`UTR;1stExon-Island-cg04720635 | 1.65 | 0.038 | 0.051 |
| TNFAIP8 - Body-Island-cg12148675 | 0.867 | 0.56 | 0.615 |
| TNFAIP8 - Body-Island-cg15292768 | 1.109 | 0.6 | 0.615 |
| TNFAIP8 - Body;TSS200-Island-cg16645133 | 0.769 | 0.25 | 0.276 |
| TNFAIP8 - Body;TSS200-Island-cg19843939 | 0.649 | 0.056 | 0.084 |
| TNFAIP8 - Body;TSS200-Island-cg24120357 | 0.671 | 0.048 | 0.060 |
| TNFAIP8 - Body;5`UTR;1stExon-Island-cg23917399 | 0.655 | 0.06 | 0.084 |
| TNFAIP8 - Body-S_Shelf-cg26305729 | 0.656 | 0.035 | 0.051 |
| STEAP4 – 5`UTR-Open_Sea-cg00564163 | 0.734 | 0.2 | 0.228 |
| STEAP4 – 5`UTR-Open_Sea-cg21227610 | 0.677 | 0.11 | 0.124 |
| STEAP4 – TSS200-Open_Sea-cg07719679 | 0.778 | 0.3 | 0.338 |
| STEAP4 – TSS200-Open_Sea-cg11271605 | 0.687 | 0.13 | 0.148 |
| STEAP4 – TSS200-Open_Sea-cg19430577 | 0.558 | 0.023 | 0.046 |
| STEAP4 – TSS200-Open_Sea-cg25783579 | 0.614 | 0.06 | 0.084 |
| STEAP4 - Body-Open_Sea-cg09379345 | 0.581 | 0.014 | 0.065 |
| STEAP4 – 3`UTR-Open_Sea-cg12308110 | 1.661 | 0.011 | 0.057 |
| STEAP4 - 5`UTR;1stExon-Open_Sea-cg12833207 | 0.594 | 0.039 | 0.060 |
| STEAP4 - TSS1500-Open_Sea-cg25143609 | 0.522 | 0.0027 | 0.028 |
| STEAP4 - TSS1500-Open_Sea-cg26619035 | 0.524 | 0.0026 | 0.028 |
| STEAP4 - TSS1500-Open_Sea-cg27636740 | 0.647 | 0.08 | 0.095 |
