# Supplementary material for: Comprehensive multi-omics analysis reveals prognostic, immune, and therapeutic signatures of TNFAIP family genes in breast cancer
Source: PLoS One. 2026 May 29;21(5):e0349012. doi: 10.1371/journal.pone.0349012 (PMC13221070; doi:10.1371/journal.pone.0349012)
Supplement: S1 Fig — The correlation between OS in BC patients and the mRNA expression of NR3C1, NFKB1, CEBPD, CEBPB and AR. P < 0.05 was the threshold for significance. The confidence intervals are shown in brackets. Black indicates low expression, whereas red indicates high expression. The x-axis indicates time (in months), and the y-axis represents survival probability. The hazard ratio is HR. (DOCX) [file pone.0349012.s004.docx]

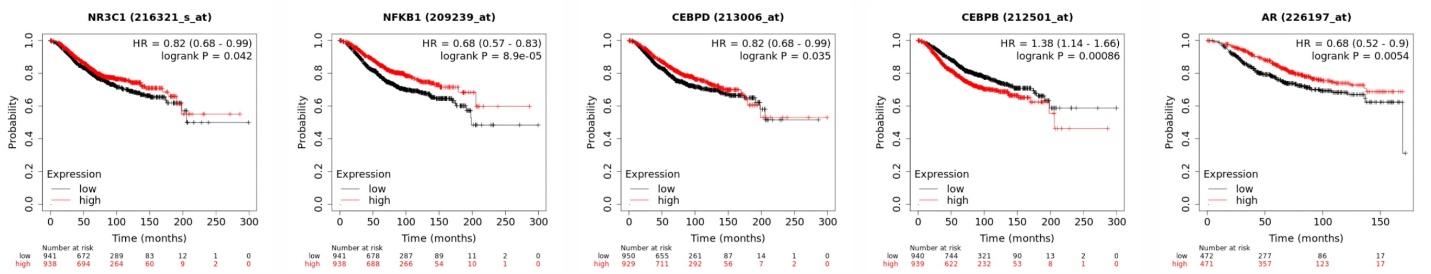


**S1 Fig** | The Kaplan-Meier plotter's prognostic value for the TFs regulating TNFAIP family members. The correlation between OS in BC patients and the mRNA expression of NR3C1, NFKB1, CEBPD, CEBPB and AR. P < 0.05 was the threshold for significance. The confidence intervals are shown in brackets. Black indicates low expression, whereas red indicates high expression. The x-axis indicates time (in months), and the y-axis represents survival probability. The hazard ratio is HR.
