# Supplementary material for: Comprehensive multi-omics analysis reveals prognostic, immune, and therapeutic signatures of TNFAIP family genes in breast cancer
Source: PLoS One. 2026 May 29;21(5):e0349012. doi: 10.1371/journal.pone.0349012 (PMC13221070; doi:10.1371/journal.pone.0349012)
Supplement: S2 Fig — The correlation between OS in BC patients and the miRNA expression of hsa-miR-23c and hsa-miR-654-5p. p < 0.05 was the threshold for significance. The confidence intervals are shown in brackets. Black indicates low expression, whereas red indicates high expression. The x-axis indicates time (in months), and the y-axis represents survival probability. The hazard ratio is HR. (DOCX) [file pone.0349012.s005.docx]

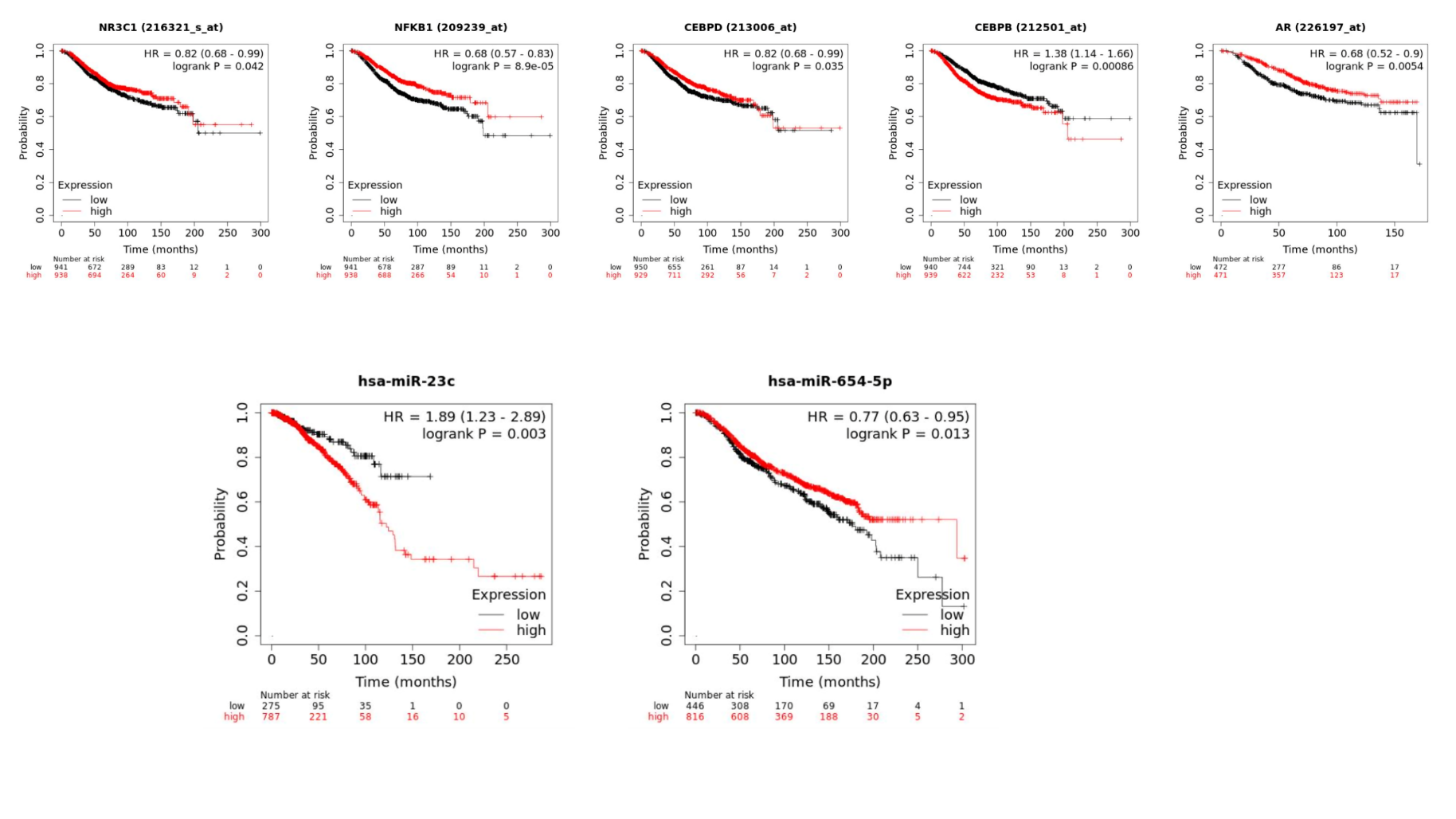


**S2 Fig** | The Kaplan-Meier plotter's prognostic value for the miRNAs regulating TNFAIP family members. The correlation between OS in BC patients and the miRNA expression of hsa-miR-23c and hsa-miR-654-5p. P < 0.05 was the threshold for significance. The confidence intervals are shown in brackets. Black indicates low expression, whereas red indicates high expression. The x-axis indicates time (in months), and the y-axis represents survival probability. The hazard ratio is HR.
