# Supplementary material for: Comprehensive multi-omics analysis reveals prognostic, immune, and therapeutic signatures of TNFAIP family genes in breast cancer
Source: PLoS One. 2026 May 29;21(5):e0349012. doi: 10.1371/journal.pone.0349012 (PMC13221070; doi:10.1371/journal.pone.0349012)
Supplement: S3 Fig — The red curve represents patients with high methylation levels, while the blue curve represents patients with low methylation levels. The x-axis indicates time (in days), and the y-axis represents survival probability. (DOCX) [file pone.0349012.s006.docx]

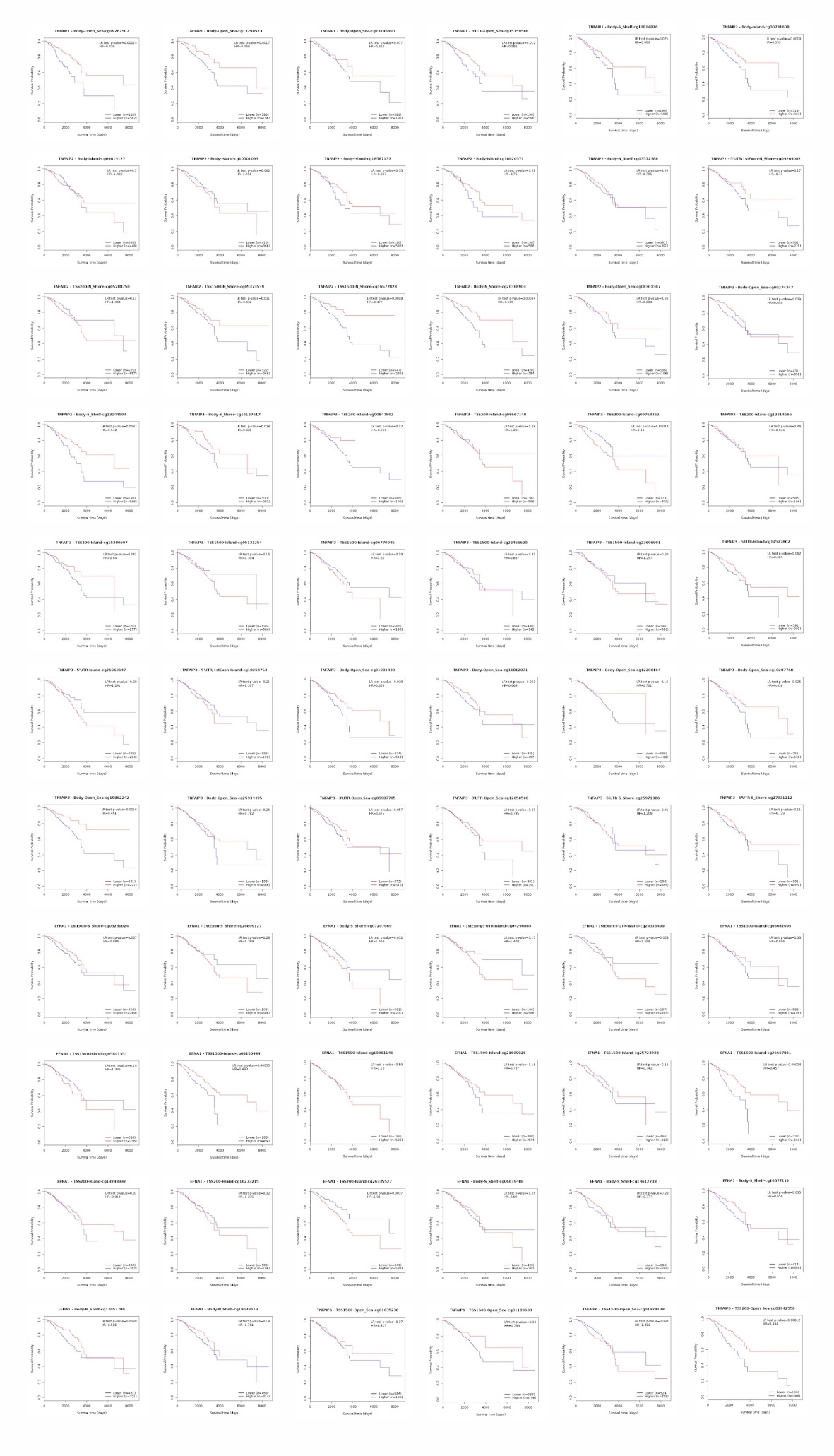


**S3 Fig** | Kaplan–Meier curves to illustrate the single CpG methylation of TNFAIP family's prognostic value in BC patients. The red curve represents patients with high methylation levels, while the blue curve represents patients with low methylation levels. The x-axis indicates time (in days), and the y-axis represents survival probability.
