# Supplementary material for: Comprehensive multi-omics analysis reveals prognostic, immune, and therapeutic signatures of TNFAIP family genes in breast cancer
Source: PLoS One. 2026 May 29;21(5):e0349012. doi: 10.1371/journal.pone.0349012 (PMC13221070; doi:10.1371/journal.pone.0349012)
Supplement: S4 Fig — This figure presents scatter plots illustrating the correlation between mRNA expression levels (log2 TPM) of TNFAIP family genes and immune cell infiltration levels in BC patients. Each subplot corresponds to a specific TNFAIP gene and evaluates the relationship with different immune cell types. The x-axis represents the infiltration level of each immune cell type, while the y-axis represents the log2 TPM expression level of the respective gene. Blue regression lines indicate the trend of correlation. Correlation coefficients (cor) and partial correlation coefficients (partial cor) are provided, with p-values indicating statistical significance. (DOCX) [file pone.0349012.s007.docx]

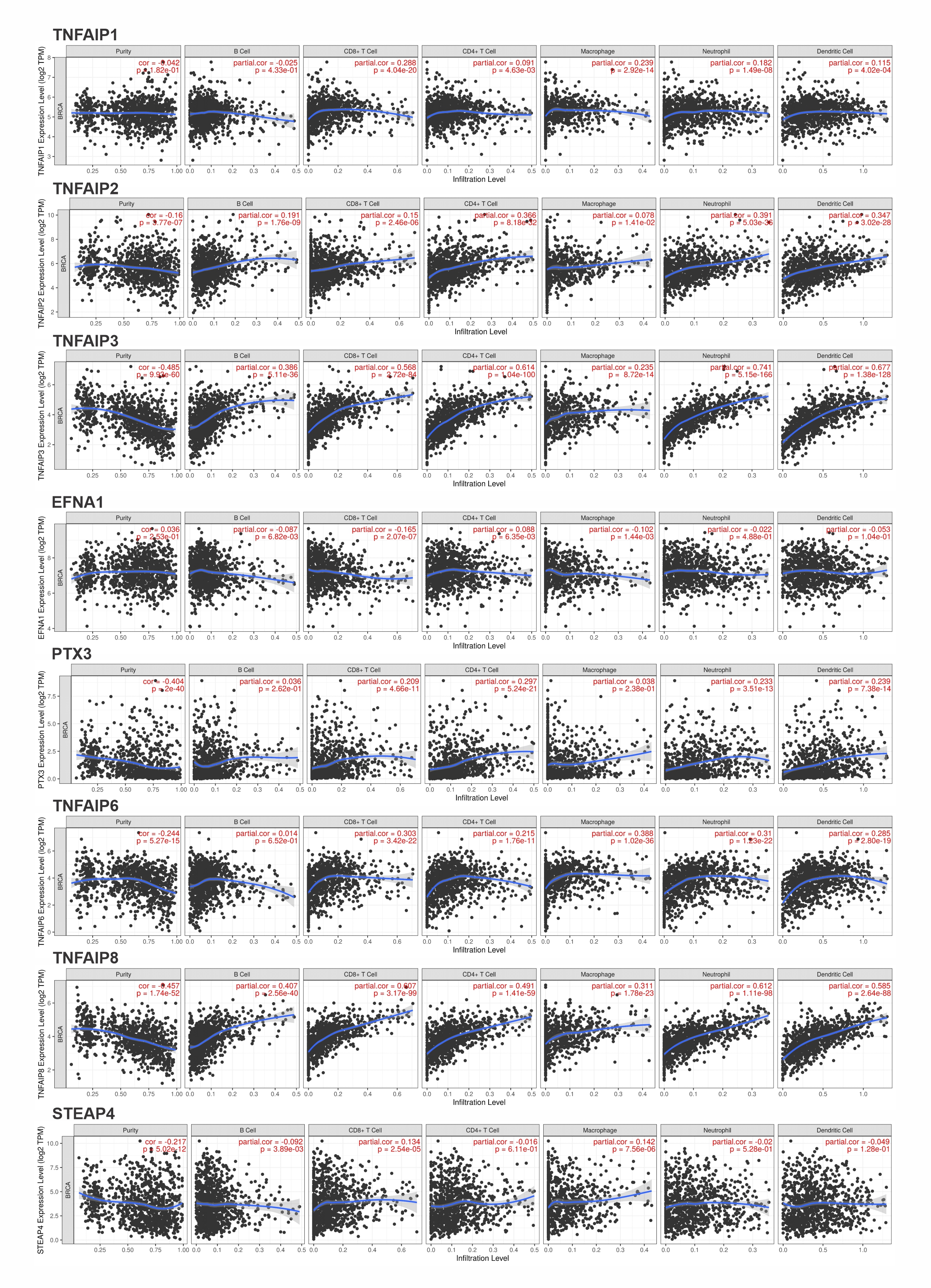


**S4 Fig** | The correlation between the expression of the TNFAIP family and the level of immune cell infiltration in BC (TIMER). This figure presents scatter plots illustrating the correlation between mRNA expression levels (log2 TPM) of TNFAIP family genes and immune cell infiltration levels in BC patients. Each subplot corresponds to a specific TNFAIP gene and evaluates the relationship with different immune cell types. The x-axis represents the infiltration level of each immune cell type, while the y-axis represents the log2 TPM expression level of the respective gene. Blue regression lines indicate the trend of correlation. Correlation coefficients (cor) and partial correlation coefficients (partial cor) are provided, with p-values indicating statistical significance.
