# Supplementary material for: Comprehensive multi-omics analysis reveals prognostic, immune, and therapeutic signatures of TNFAIP family genes in breast cancer
Source: PLoS One. 2026 May 29;21(5):e0349012. doi: 10.1371/journal.pone.0349012 (PMC13221070; doi:10.1371/journal.pone.0349012)
Supplement: S5 Fig — A: A bubble plot was used to illustrate the relationship between the expression of the TNFAIP family and the sensitivity of the top GDSC drugs in pan-cancer. B: A bubble plot was used to illustrate the relationship between the expression of the TNFAIP family and the sensitivity of CTRP drugs (top 30) in pan-cancer. The x-axis represents different drugs, while the y-axis lists TNFAIP family genes. The color intensity and direction indicate the correlation strength and direction: purple represents negative correlation (up to −0.4), while red represents positive correlation (up to 0.5). The size of the circles reflects the False Discovery Rate (FDR) significance levels. (GDSC: Genomics of Drug Sensitivity in Cancer, CTRP: Cancer Therapeutics Response Portal, GSCALite: Gene Set Cancer Analysis). (DOCX) [file pone.0349012.s008.docx]

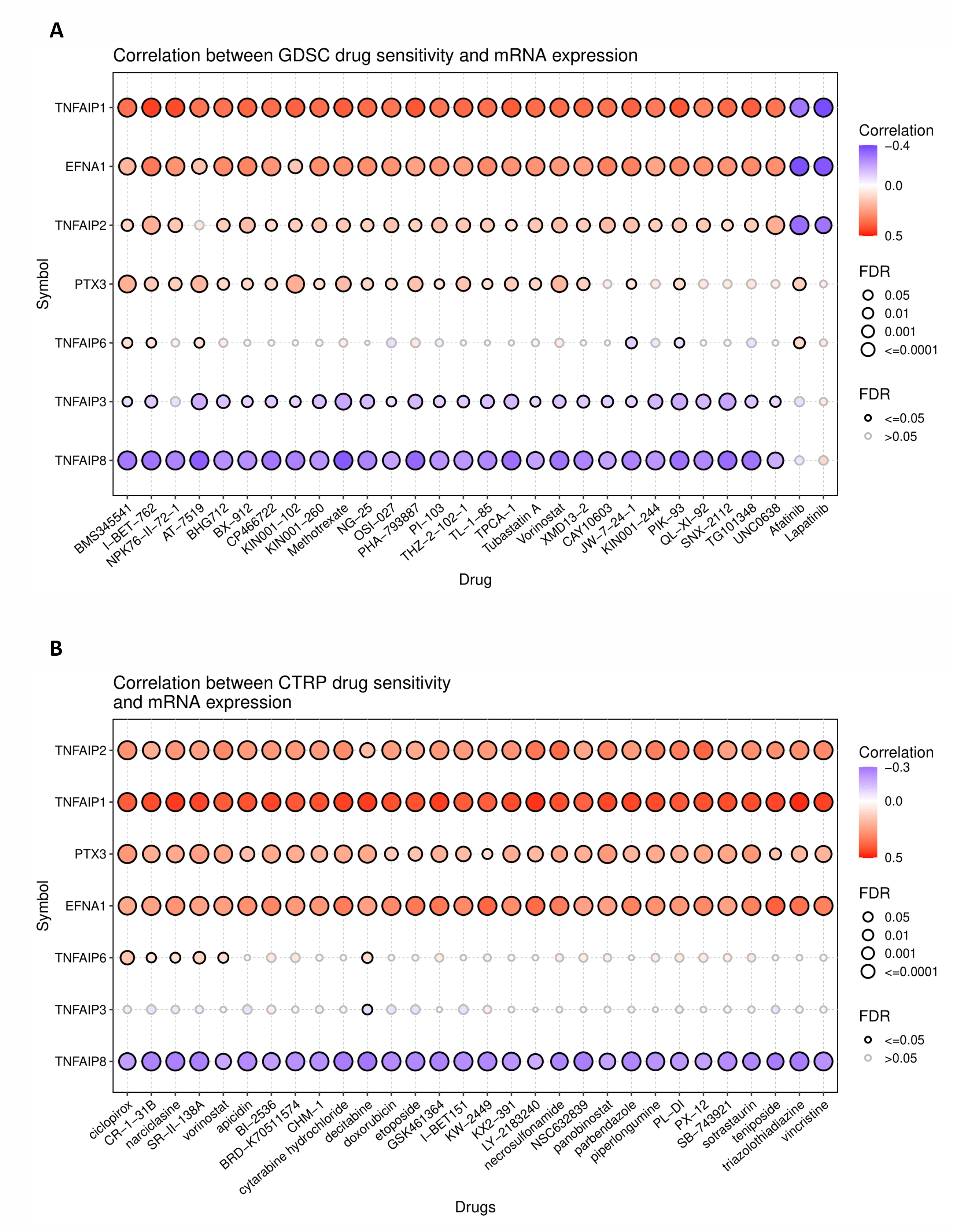


**S5 Fig |** Association between TNFAIP family and sensitivity to FDA-approved drugs (GSCALite database). **A**: A bubble plot was used to illustrate the relationship between the expression of the TNFAIP family and the sensitivity of the top GDSC drugs in pan-cancer. **B**: A bubble plot was used to illustrate the relationship between the expression of the TNFAIP family and the sensitivity of CTRP drugs (top 30) in pan-cancer. The x-axis represents different drugs, while the y-axis lists TNFAIP family genes. The color intensity and direction indicate the correlation strength and direction: purple represents negative correlation (up to -0.4), while red represents positive correlation (up to 0.5). The size of the circles reflects the False Discovery Rate (FDR) significance levels. (GDSC: Genomics of Drug Sensitivity in Cancer, CTRP: Cancer Therapeutics Response Portal, GSCALite: Gene Set Cancer Analysis)
