# Supplementary material for: Comprehensive multi-omics analysis reveals prognostic, immune, and therapeutic signatures of TNFAIP family genes in breast cancer
Source: PLoS One. 2026 May 29;21(5):e0349012. doi: 10.1371/journal.pone.0349012 (PMC13221070; doi:10.1371/journal.pone.0349012)
Supplement: Attachment — Submitted filename: Response_to_Reviewers_auresp_3.docx [file pone.0349012.s012.docx]

**Response to reviewer**

Submit number: PONE-D-25-56610R2

Article title: Comprehensive multi-omics analysis reveals prognostic, immune, and therapeutic signatures of TNFAIP family genes in breast cancer

**Comment:** The authors addressed all the comments & feedback from the reviewers sensibly.However, a link (http://bioinfo.life.hust.edu.cn/web/GSCALite/) to one of the tools used (GSCALite) is broken, which seems to have a working link on GitHub (https://github.com/chunjie-sam-liu/GSCALite).

Can the authors please revise their manuscript and correct this issue with the broken link?

**Response:** Thank you for pointing out the issue regarding the broken link in our manuscript. We have now corrected the broken link for GSCALite in our manuscript. As requested, we have replaced the previous URL with the active GitHub link (<https://github.com/chunjie-sam-liu/GSCALite>).

We have uploaded the revised files as requested:

1. Response to Reviewers: Containing this note.

2. Revised Manuscript with Track Changes: Showing the updated link.

3. Manuscript: The clean version without track changes.

We appreciate your patience and your guidance in helping us improve our manuscript. We look forward to hearing from you soon.
